# Blood-Brain Barrier Breakdown Following Traumatic Brain Injury: A Possible Role in Posttraumatic Epilepsy

**DOI:** 10.1155/2011/765923

**Published:** 2011-02-22

**Authors:** Oren Tomkins, Akiva Feintuch, Moni Benifla, Avi Cohen, Alon Friedman, Ilan Shelef

**Affiliations:** ^1^Departments of Physiology and Neurobiology, Zlotowski Center for Neuroscience, Ben-Gurion University of the Negev, 84105 Beer-Sheva, Israel; ^2^Department of Ophthalmology, Bnai Zion Medical Center, Haifa, Israel; ^3^Department of Neuroradiology, Soroka University Medical Center and Zlotowski Center for Neuroscience, Ben-Gurion University of the Negev, Beer-Sheva, Israel; ^4^Department of Neurosurgery, Soroka University Medical Center and Zlotowski Center for Neuroscience, Ben-Gurion University of the Negev, Beer-Sheva, Israel; ^5^Department of Biomedical Engineering, Ben-Gurion University of the Negev, Beer-Sheva, Israel

## Abstract

Recent animal experiments indicate a critical role for opening of the blood-brain barrier (BBB) in the pathogenesis of post-traumatic epilepsy (PTE). This study aimed to investigate the frequency, extent, and functional correlates of BBB disruption in epileptic patients following mild traumatic brain injury (TBI). Thirty-seven TBI patients were included in this study, 19 of whom suffered from PTE. All underwent electroencephalographic (EEG) recordings and brain magnetic resonance imaging (bMRI). bMRIs were evaluated for BBB disruption using novel quantitative techniques. Cortical dysfunction was localized using standardized low-resolution brain electromagnetic tomography (sLORETA). TBI patients displayed significant EEG slowing compared to controls with no significant differences between PTE and nonepileptic patients. BBB disruption was found in 82.4% of PTE compared to 25% of non-epileptic patients (*P* = .001) and could be observed even years following the trauma. The volume of cerebral cortex with BBB disruption was significantly larger in PTE patients (*P* = .001). Slow wave EEG activity was localized to the same region of BBB disruption in 70% of patients and correlated to the volume of BBB disrupted cortex. We finally present a patient suffering from early cortical dysfunction and BBB breakdown with a gradual and parallel resolution of both pathologies. Our findings demonstrate that BBB pathology is frequently found following mild TBI. Lasting BBB breakdown is found with increased frequency and extent in PTE patients. Based on recent animal studies and the colocalization found between the region of disrupted BBB and abnormal EEG activity, we suggest a role for a vascular lesion in the pathogenesis of PTE.

## 1. Introduction

Traumatic brain injury (TBI) is a common cause of mortality and morbidity with an occurrence of approximately 200 cases per 100,000 people a year. It is also a known major risk factor for focal epilepsy [1]. The incidence of post-traumatic epilepsy (PTE) ranges from 2–50% in different studies, accounting for approximately 20% of symptomatic epilepsies [1–6]. Seizures may occur immediately following the trauma, though PTE usually develops several months and even years later. While immediate post-traumatic seizures may be successfully treated with antiepileptic drugs [7], the mechanisms underlying the development of PTE remain unknown with no means for preventing it [8].

The central nervous system is protected by the function of the blood-brain barrier (BBB), which regulates the passage of blood constituents in and out of the brain extracellular space. It has been previously suggested that an increase in BBB permeability may be associated with the pathogenesis of neurological disorders [9–11]. However, only recent animal experiments directly showed that primary prolonged opening of the BBB leads to the development of delayed, long-lasting epileptiform activity [12]. Furthermore, it has been suggested that the most common serum protein, albumin, may underlie astrocytic activation and dysfunction, further leading to neuronal hypersynchrony (i.e., *epileptogenesis*) and accumulated neuronal loss [13, 14]. While previous clinical studies showed that altered permeability is observed in neurological patients [15–18], little data exists on the frequency, extent and significance of enhanced BBB permeability in epileptic patients [19]. The aim of the present study was to characterize the frequency of long-lasting increases in BBB permeability following head trauma and explore correlations with the extent of cerebral lesions, EEG abnormalities, and the presence of post-traumatic epilepsy. Part of this study has been published as a short report [20].

## 2. Materials and Methods

### 2.1. Patient Selection

The study protocol was approved by the Soroka Medical Center Medical Ethics Board and Helsinki Committee (NIH clinical trial registration: NCT00419874). Patients were included in the study if they were referred to the tertiary center's outpatient clinic following hospitalization due to TBI, most often with significant symptoms. Thirty-seven head trauma patients (11 women, 26 men) aged 10–68 years (26.89 ± 2.43), who were examined during the years 2005–2007 were included in this study (Table 1). Patients were included if, at the time of enrollment, they were more than one week after TBI (28.81 ± 8.81, median= 3 months), were fully conscious, and with mild to no neurological impairment. All patients were healthy prior to the traumatic brain injury with no history of neurological or psychiatric disorders. Eighteen patients had been involved in moving vehicle accidents, 7 had fallen, and 12 were hit by a blunt instrument (hammer, door, fist, etc.). Most patients (*n* = 34, 91.89%) suffered from mild TBI according to a Glasgow coma score (GSC) of >13 upon admission (in 88% the documented score was 15). A short period of unconsciousness lasting up to several minutes was noted in 13 patients, and 3 patients suffered from loss of consciousness lasting several days. In the 21 remaining patients, no period of unconsciousness was reported. All patients made a good recovery before being enrolled in the study. 

Nineteen patients were diagnosed as suffering from PTE with partial seizures (in 11 patients, secondary generalization was reported). A diagnosis of PTE was given to patients that presented with at least one delayed epileptic seizure (more than a week after the trauma). The non-epileptic TBI patients (*n* = 18) mainly suffered from headaches (*n* = 15, 83.33%), cognitive impairment (*n* = 2, 11.11%), mild motor dysphasia (*n* = 1, 5.56%), or an acute stress reaction (*n* = 1, 5.56%). Both the PTE and non-epileptic groups were similar in age (27.05 ± 3.78 and 26.72 ± 3.14 years, resp., *P* = .95) and gender (7 women and 12 men, and 4 women and 14 men, resp., *P* = .33). A third group, consisting of 13 healthy adult volunteers (5 women and 8 men, aged 34.77 ± 2.47 years) with no history of brain injury or neurological disease, served as a control for the quantitative electroencephalography (qEEG) studies. A fourth group of 8 healthy adult volunteers (3 women and 5 men, aged 29.57 ± 0.48) underwent bMRI scans for normal BBB function measurements.

### 2.2. Quantitative Electroencephalography

qEEG recordings were carried out using a clinical 128 channel digital EEG acquisition unit (CEEGRAPH IV, Bio-logic Systems Corp., Mundelein, Illinois), with a digitization rate of 256 Hz. Twenty-three conventional AgCl surface electrodes were placed according to the international 10–20 electrode system, with additional electrodes placed at both ear lobes. Scalp electrode impedances were kept below 10 kΩ. The band pass was set at 0.1 to 100 Hz. EEG data was visually inspected, and 50–80 seconds of artifact-free, closed eye data were extracted for quantitative analysis. Fast Fourier transform (FFT) was applied to the EEG waveforms recorded from each electrode of each subject. A periodogram was used to calculate the average power spectrum. The EEG was then clinically interpreted by a physician unaware of the study. For each subject, the average value for the discrete frequency bands (delta 1.5–4 Hz, theta 4.5–7.5 Hz, alpha 8–12 Hz, beta 13–30 Hz) was normalized to each subject's own total power of the 1.5–40 Hz frequency spectrum (values are represented as % of the total electrode power).

### 2.3. Magnetic Resonance Imaging

MRI scans were performed using a 1.5 Tesla machine (Intera, Philips Medical Systems, Best, the Netherlands). For BBB integrity evaluations, images were collected before and following the peripheral administration of the contrast medium Magnetol® (Gadolinium-DTPA (Gd-DTPA) 0.5 M, 0.1 mmol/kg) (Soreq Radiopharmaceuticals, Israel), as described below.

### 2.4. BBB Integrity Evaluation

Two independent methods were used in the present study to estimate BBB permeability: (1) a semiquantitative method was used to detect and calculate the volume of BBB disrupted cortex; (2) a dynamic method was used for measuring the relative change in BBB disrupted volume with time. 

#### 2.4.1. The Semi-Quantitative Evaluation of BBB Permeability

**Figure 1:**
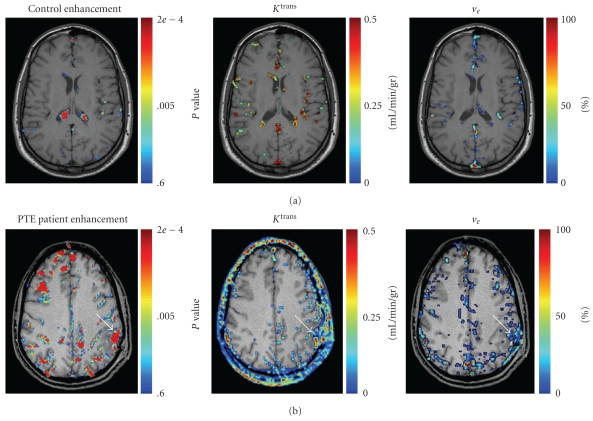
Imaging modalities allow for BBB permeability evaluation and quantification. (a) Control subject: no BBB disruption is observed within the brain parenchyma (areas of significant differences are found in blood vessels, sinuses and the choroid plexus). Increased BBB permeability (*K* ^trans^) and extravascular volume (*v*_*e*_) are also localized to those same structures. (b) A 28-year-old PTE patient 10 days following TBI. A region of increased BBB permeability is detected over the left parietal lobe in both methods (arrows).

(Figure 1(a))—Axial T1-weighted spin-echo images were obtained (582/15/1 [TR/TE/NEX], section thickness, 5 mm; intersection gap, 1 mm; matrix, 256 × 256) were performed before and following the peripheral addministration of Gd-DTPA. Using a manual anatomical landmark identification method [21], we paired matching brain images before and after the administration of Gd-DTPA. Image analysis was performed on matching images in the dicom format. Using a field of view of 230 × 230 mm resulted in a resolution of 0.81 mm^2^/pixel, or 12.92 mm^2^/population group. Matching populations were compared to detect changes in signal intensity. These were inspected for either differences in percent enhancement (pertaining to the presence of the contrast agent), or for statistical significance (using Student's *t*-test). In order to detect abnormal penetration of contrast agent through the BBB, we set the “detection threshold" to >20% increase in signal intensity (i.e., >2 standard deviations from the normal parenchymal mean). The Bonferroni correction for multiple simultaneous statistical tests was applied for the statistical calculations. Changes in signal enhancement were considered to be due to BBB disruption if they occurred within the brain parenchyma. 

#### 2.4.2. Dynamic Evaluation of BBB Permeability

(Figure 1(b))—The imaging protocol for this contrast-enhanced studies was modified according to published methods [22]. Four consecutive 3D RF spoiled T1 weighted field echo acquisitions with an array of flip angles (*α* = 2°, 10°, 20°, 35°) were performed to allow calculation of T1 maps. The third sequence was then repeated (*n* = 80) to produce a T1 weighted dynamic data set with a time resolution of 5.5 seconds and a duration of approximately 10 minutes. Contrast agent was given as an intravenous bolus injection over a period of 4 seconds following the fifth dynamic scan. Maps of proton density *M*_0_ and intrinsic longitudinal relaxation rate (*R*10 = 1/*T*10) were calculated. 4D (*x*, *y*, *z*, *t*) postinjection longitudinal relaxation rate [*R*1(*t*)] maps were calculated for each T1 weighted dynamic phase using signal intensity data from pre- and postcontrast T1 field echo images [*S*(*t*) − *S*(0)]. 4D Gd-DTPA concentrations [*C*(*t*)] maps were then calculated from the 4D *R*1(*t*) maps:

(1) $$C\left(t\right)=\frac{\left(R1\left(t\right)\;-R10\right)}{r_{1}},$$

where *r*1 is the relaxivity of Gd-DTPA determined experimentally. *r*_1_ = 4.39 [s^−1^mM^−1^] (at 37°C and at 1.5 T). The time course of the intravascular contrast concentration was used to calculate an effective vascular input function (VIF). Maps of *R*10, *C*(*t*), and the VIF were then used to calculate the volume transfer constant between blood plasma and extravascular extracellular space (EES)—*K* ^trans^, and the volume of extravascular extracellular space per unit volume of tissue—*v*_*e*_, on a pixel by pixel basis using a standard compartmental model. Using this model, the time course of contrast agent concentration in tissue can be described by the following equation:

(2) $$C_{t}\left(t\right)=K^{\text{trans}}\left(C_{p}\left(t\right)⊗\exp\;\left(-k_{\text{ep}}\cdot t\right)\right)$$

*C*_*t*_(*t*) represents the tracer concentration in the tissue at time *t*, *C*_*p*_(*t*) the tracer concentration in blood plasma at time *t*, *k*_ep_= *K* ^trans^/*v*_*e*_ the rate constant between EES and blood plasma and ⊗ denotes convolution. Resulting images represent BBB permeability (defined as *K* ^trans^[min^−1^] = PS*ρ* where PS[mL min^−1^ gr^−1^] is the permeability of a surface area product per unit mass of tissue, and *ρ* [g ml^−1^] is the density of tissue) and the volume fraction of the EES (expressed as the volume of contrast agent per volume of pixel, and therefore represented as percentage).

Normalization of the quantified permeability values was performed to the average value of the contralateral uninvolved hemisphere.

### 2.5. Calculating Lesion and BBB Disrupted Volume and Location

Measuring the size of the cortical lesion and BBB disrupted area was performed using MATLAB (version 7.1) on images obtained through the semi-quantitative evaluation. For lesion volume measurements, T1 MRI images before contrast agent administration were used. An experienced neuroradiologist identified the location of the parenchymal lesion in all slices, and the number of pixels was counted. BBB disruption volume was performed on the same slices where the lesion was identified. Using the signal enhancement results for those slices, the number of pixels with enhancement above 20% was counted. Lesion and BBB-disrupted volumes are displayed in cm^3^. 

For quantifying BBB permeability, values of all pixels within a region of interest were collected using the dynamic method and a frequency distribution was calculated. The extravascular volume of the contrast agent (*v*_*e*_) was quantified by calculating the sum of values within the region of interest. Localization of the cortical and BBB lesions according to Brodmann areas was performed by manual anatomical registration to the digitized Talairach brain atlas.

### 2.6. Statistical Analysis

The nonparametric Mann-Whitney U test was used for evaluating statistical significance of the differences in the power spectrum between controls and the patient groups, the change in cortical lesion and BBB disrupted volumes compared to neuronal dysfunction as measured by qEEG, and for changes in permeability, extravascular volume, and delta band power with time. The *χ* ^2^ Pearson's test was used for evaluating the frequency of abnormal qEEG activity, and cortical lesions between the PTE and non-epileptic groups. Correlations between BBB disruption or lesion size and the volume of dysfunctional cortex were performed using the Student's *t*-test. All results are presented as mean ± SEM.

## 3. Results

### 3.1. Lasting BBB Disruption in TBI Patients

30 patients underwent bMRI scans (15 PTE and 15 non-epileptic) and the images were evaluated for parenchymal lesions and disruption volumes. In the healthy control group (*n* = 8), significant enhancement was only observed in blood vessels and regions known to lack a BBB (Figure 1(a) and also see [15]). In contrast, 16 TBI patients (53.3%) showed parenchymal regions with enhanced signal indicating BBB disruption (Figure 1(b)). In 15 of these patients (93.8%) the disrupted BBB was located in cortical regions surrounding old contusions (and with no involvement of more distant cortical regions), suggesting a local trauma-related mechanism. In a single patient BBB disruption was detected in a parietal region, but no concomitant cortical lesion was found in any sequences including T2*. 

BBB disruption was identified up to several months following the traumatic event (19.4 ± 9.4, median = 2.5 months), with a delay of 1.5–11 years in 4 patients. Cortical lesions were found in the parietal (*n* = 11, 68.8%), temporal (*n* = 3, 18.8%), frontal (*n* = 1, 6.3%), and occipital (*n* = 1, 6.3%) regions. The average lesion volume was 6.0 ± 1.7 cm^3^ and the average volume of cortex with disrupted BBB was 5.9 ± 1.6 cm^3^. Although PTE patients were more likely to have a lesion diagnosed on their MRI scans than non-epileptic patients (83.3 versus 38.9%, *P* = .006), there was no significant difference with respect to the size of the lesion (6.6 ± 1.9 versus 5.3 ± 2.8 cm^3^, *P* = .19). Conversely, PTE patients were more likely to have BBB disruption than non-epileptic patients (82.4 versus 25%, *P* = .001), and the volume of BBB disruption was significantly larger (9.8 ± 2.6 versus 1.7 ± 0.6 cm^3^, *P* = .001).

### 3.2. EEG Analysis in Post-Traumatic Patients

**Figure 2:**
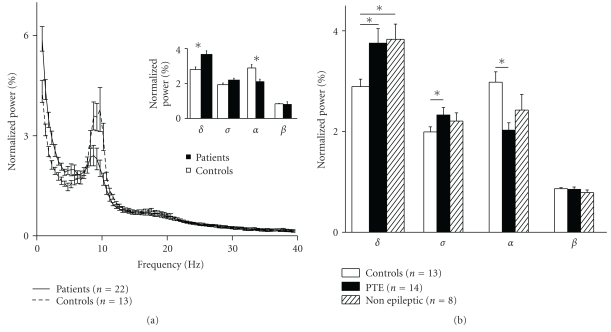
Abnormal qEEG in TBI patients. (a) Average normalized power spectrum representation of all TBI patients and controls that underwent qEEG. Note the significant increase in delta power and the decrease in the alpha band in TBI patients compared to controls (inset). (b) Power spectrum averages of patient subgroups according to the occurrence of seizures. While both the PTE and the non-epileptic group had elevated delta power compared to controls, only PTE patients had a significant reduction of alpha and increase of theta power. *= *P* < .05.

EEG recordings were performed on 22 TBI patients 10 days–11 years (median= 3 months) after the trauma. Abnormal EEG slowing or interictal epileptiform activity was detected in 78.6% (11 of 14) of the PTE patients and in 12.5% (1 of 8) of the non-epileptic group (*P* = .006). Comparison of FFT spectral analysis between the TBI patients and the healthy control group revealed a significant increase in delta power among the TBI patients (3.7 ± 0.2 versus 2.8 ± 0.2 %, for patients vs. controls, resp.; *P* = .002) and a significant decrease in the alpha band power (2.1 ± 0.1 versus 2.9 ± 0.2 %, *P* = .005, Figure 2(a)). The significant increase in delta power was similar in both the PTE (3.68 ± 0.28 %) and non-epileptic patients (3.76 ± 0.29 %). In contrast, only the PTE group showed a significantly reduced alpha (1.99 ± 0.14 and 2.83 ± 0.15 %, *P* = .01) and elevated theta power (2.28 ± 0.14 and 1.93 ± 0.09 %, *P* = .04, Figure 2(b)) compared to the controls.

### 3.3. BBB Breakdown and Source Localization of Abnormal EEG Patterns

**Figure 3:**
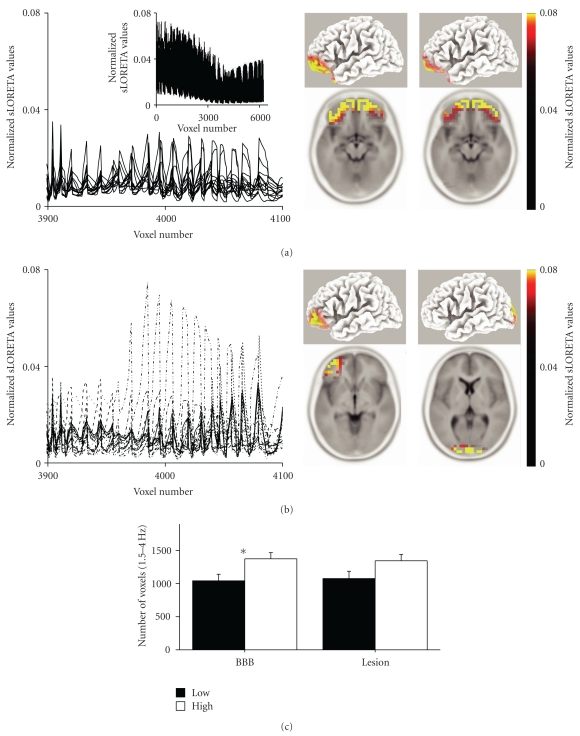
The extent of qEEG slowing in PTE patients correlates with BBB disruption. (a) An enlarged region of normalized sLORETA values for the delta band in voxels number 3900–4100 from healthy controls. The entire normalized sLORETA for the delta band is shown in the inset, as are 2 examples of signal localization to the frontal midline region. (b) The PTE population displayed marked variability among the same voxels, and maximal signal localization was varied according to the site of injury. (c) The volume of cortex with abnormal cortical activity according to sLORETA between patients with the bottom (black) and top (white) half volume of BBB disruption or cortical lesion. Note that patients with a larger volume of BBB disruption also had a significantly larger volume of dysfunctional cortex. *= *P* < .05.

The spatial relationship between BBB disruption and abnormal cortical function was assessed by localizing the cortical sources of pathological slow delta activity using sLORETA. In healthy individuals (control group), the voxels generating delta activity were consistently localized to medial frontal and interhemispheric cortical structures (Figure 3(a)). In contrast, TBI patients displayed a high variability for the maximal activity region (Figure 3(b)). 10 TBI patients with identified BBB disruption underwent EEG recordings. In 7 of them, sLORETA localized the source for abnormal delta to the same Brodmann area as that of the BBB disruption. 

The volume of cortex with slow delta activity was estimated by counting the number of voxels one standard deviation away from the average control value. This was significantly greater among TBI patients with a large BBB disrupted lesion than patients with small lesions (1042.6 ± 5.3 versus 1373.6 ± 4.13 voxels, *P* = .03, Figure 3(c), suggesting a close relationship between increased BBB permeability and cortical dysfunction. No significant difference was found in relation to the size of the anatomical cortical lesion (1075.7 ± 6 versus 1344.6 ± 4.3 voxels, *P* = .09). 

### 3.4. BBB Breakdown and EEG Followup with Time: A Case Description

**Figure 4:**
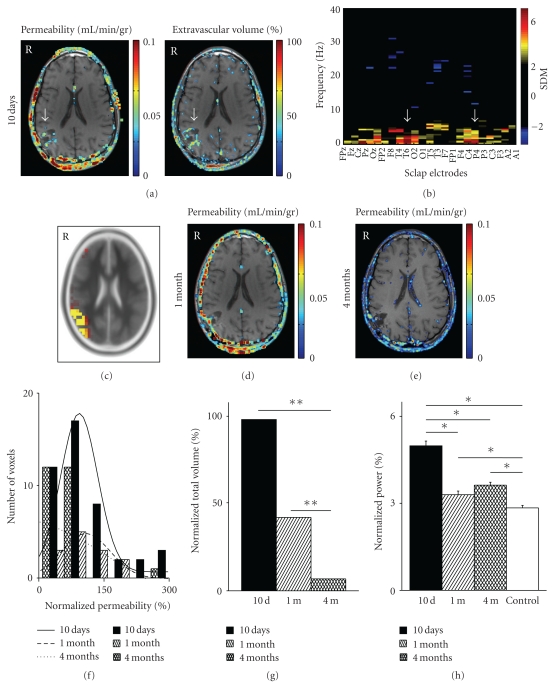
Abnormal EEG slowing localizes to region of BBB disruption in a 15-year-old PTE patient one month following mild TBI (see text for details). (a) BBB evaluation 10 days after the event revealed a focal area of increased BBB permeability (left, arrow, Brodmann area 40), surrounded by an increase of extravascular volume (right, arrow). (b) Representation of the EEG recording with the x-axis representing the 23 different electrodes, the y-axis the frequencies at 0.5 Hz intervals, and the colour coding the number of standard deviations from the average control EEG. Note the increased power in the delta range over the right temporoparietal electrodes (arrows). (c) sLORETA localized the delta activity to the right parietal region (Brodmann area 40). Repeated MRI scans 1 (d) and 4 months (e) following the trauma revealed a resolution of the BBB lesion. (f) Histogram representation of the permeability values surrounding the cortical lesion (normalized to the average value of the contralateral hemisphere). (g) Four months after the trauma there is a significant decrease in the permeability values, as well as in the extravascular volume. (h) Quantification of the average delta power shows a significant reduction in delta wave activity as time progresses, though remaining significantly increased compared to controls even 4 months after the event and resolution of the BBB lesion. * = *P* < .05, ** = *P* < .0001.

In three patients, we performed repeated bMRI and EEG studies to underscore the dynamic relations between abnormal BBB permeability and cortical dysfunction. One such patient with post-traumatic BBB opening and PTE is presented here in more detail (Figure 4): a 15-year-old boy was admitted two hours after a blunt head injury during a moving vehicle accident, causing a short (minutes) episode of unconsciousness. Upon admission, he was conscious and a full neurological examination was normal (GCS = 15). A brain computed tomography (bCT) scan showed an open depressed right parietal skull fracture, and a right parietal subarachnoid hemorrhage with no mass effect. The depressed fracture was elevated under general anesthesia. The patient was discharged with no complications on day 5. A repeated bCT scan that was performed on day 7 revealed absorption of the hemorrhage with good positioning of the fractured bone. Ten days after the trauma he had a partial seizure consisting of involuntary movements on his left hand and was therefore readmitted. A bMRI scan revealed a small right parietal hemorrhagic contusion. Dynamic BBB analysis detected increased permeability with a larger area of extravascular permeation around the contused brain (Figure 4(a)). qEEG recordings showed increased slow wave activity (1–6 Hz) over the right temporoparietal electrodes (Figure 4(b)). Interictal spikes were observed during his recording and localized to the right temporo-parietal region by sLORETA (Brodmann area 40, Figure 4(c)). He was released under medical treatment with Sodium Valporate. Subsequent MRI scans and qEEG recordings (performed on the same day, ca. 24 hours after previous antiepileptic medication) were conducted one and four months later. MRI scans revealed a persistent, though smaller area of BBB disruption over the same region (Figures 4(d) and 4(e)). Quantification of the imaging data revealed that the pattern of the normalized permeability shifted towards lower permeability values between day 10 and four months (*P* < .0001, Figure 4(f)). In parallel, the contrast agent extravascular volume also diminished, reflecting a smaller area of BBB disruption (*P* < .0001, Figure 4(g)). Quantitative EEG evaluation showed that the power of the delta band similarly diminished with time. It is interesting to note that 4 months after the trauma, while the extravascular volume of the contrast agent returned to control values (compared to the contralateral hemisphere) the delta power remained significantly higher than that of the control population (*P* = .007, Figure 4(h)—see below). 

## 4. Discussion

In this study, we investigated anatomical and functional characteristics from symptomatic patients following mild to moderate head trauma. In 37 TBI patients, we used two different quantitative methods for evaluating BBB permeability as well as EEG spectral analysis with a source localization method. We found that TBI patients show (1) a lasting focal increase in BBB permeability in up to 70% of TBI patients; (2) increased EEG slowing (compared to healthy controls) which seems to originate from a focal cortical region; (3) a good spatial correlation between the BBB lesion and the presumed source for abnormal neuronal (qEEG) activity; (4) a correlation between the size of the BBB disrupted region, but *not* the anatomical lesion, with the extent of neuronal dysfunction, and (5) patients with PTE have a higher likelihood to show abnormal BBB permeability and of a larger cortical area compared to post-traumatic patients without epilepsy. It is important to note that our study suffers from a significant “selection bias" as patients were recruited from a tertiary outpatient clinic seeing patients that were hospitalized due to TBI and referred due to significant symptomatology. Therefore, they do not reflect the true prevalence of PTE in this patient population. The relatively high rate of BBB disruption noted in our study may also reflect this selection bias. In addition, the use of our quantitative imaging methods may have increased the sensitivity of BBB breakdown measurements which would not be identified using other methods.

### 4.1. Cortical Dysfunction in TBI Patients

In accordance with earlier studies [16, 23–25], we find that patients with mild to moderate TBI commonly have abnormal EEG recordings. Interestingly, although PTE patients were more likely to have interictal sharp activity on their EEG than non-epileptic post-traumatic patients, quantitative analyses did not reveal significant differences between the two groups. EEG slowing most likely reflects dysfunction of the cortical network and neuronal hypersynchronization [26]. Indeed, while different animal models for brain injury consistently revealed electrophysiological evidence for neuronal hyperexcitability and hypersynchronicity [12–14, 27–30], only rarely have behavioral manifestations of seizures been reported [31, 32]. This stresses the well-known observation that neuronal hypersynchrony (and slowing) does not always manifest clinically as convulsions [33], probably depending on the cortical region involved. The lack of differences found between qEEG analysis in our PTE and non-epileptic symptomatic patients may arise from the fact that these groups indeed share a common path, that is, very similar pathologic neuronal dysfunction presenting with different phenotypes. Further research into the differences between these groups is needed to address this issue. 

### 4.2. BBB Disruption May Lead to Cortical Dysfunction

Recent work has shown that abnormal slow wave cortical activity following traumatic head injury can be localized to a focal region related to the site of trauma [16, 34, 35]. Considering that an average calculation of the delta band amplitude from all electrodes may yield little information (since the source of abnormal activity is not equally distributed in all scalp electrodes, but related to the site of injury), we used sLORETA to calculate the distribution of the delta band in 6239 voxels representing the entire cerebral cortex gray matter. As expected, there was a high variability in EEG current density distribution in PTE patients when compared to healthy controls, probably reflecting focal, trauma-related brain dysfunction (Figure 3). sLORETA localized the site of maximal delta wave activity in close proximity to the MR-defined cortical lesion, suggesting a causative relationship. This implies that EEG slowing is probably due to focal cortical generators (which differ in location between patients) rather than a single common “pathological” generator or general diffuse cortical slowing, as one would expect in the case of a general stress-dependent mechanism or lesion in deeper brain structures like the thalamus or brain stem [34, 35]. Although a correlation was found between the size of the BBB lesion and the volume of cortical dysfunction, no such relationship was found with the size of the anatomical lesion. This may reflect the fact that following trauma, the contused brain starts undergoing a rapid process of neuronal necrosis and gliosis and therefore is not the source of abnormal neuronal activity. However, the surrounding nonnecrotic tissue remains functional and may be exposed to abnormal conditions that impact on its normal neuronal behavior. This implies that other unknown trauma-related processes may be at the basis of cortical dysfunction. Indeed, animal studies show that cortical hyperexcitability develops in regions surrounding the site of direct trauma [36–38]. 

Based on the following observations, this study supports the supposition that lasting BBB disruption is related to the emergence of neocortical dysfunction: (1) both BBB disruption and the estimated source of pathological delta wave activity were colocalized in the cerebral cortex. (2) The greater the volume of BBB disruption the larger the area of cortical dysfunction. (3) In select cases, BBB disruption and cortical dysfunction resolve simultaneously. We cannot however rule out that abnormal neural activity causes alterations in the permeability of the local vascular bed, or that both result directly from trauma but by different mechanisms. The observation that increased BBB permeability is noted in some patients despite apparently complete medical control of seizures supports the hypothesis that the vascular pathology is not a direct result of the pathological neuronal discharge. Our hypothesis is also strongly supported by recent animal experiments demonstrating that BBB breakdown or direct exposure of the cerebral cortex to serum albumin induces activation of astrocytes, reduced buffering of extracellular potassium leading to neuronal hyperexcitability [13]. Perfusion MRI studies were not performed as part of our routine protocol, thus we cannot entirely exclude increased local cerebral blood flow as an underlying or additional source for the observed enhancement. However, this is unlikely, since no enhancement is usually observed in patients with focal epilepsy from other causes (data not shown). 

Finally, this preliminary study calls for prospective human studies which are required to elucidate the prevalence of BBB breakdown in TBI patients using sensitive imaging modalities and the possible causative relationship between early breakdown of the BBB and the development of PTE.

## 5. Conclusion

Our study, for the first time, shows that quantitative and repeated measurements of BBB permeability in human patients are possible using routine imaging techniques with postprocessing. This provides powerful tools for evaluating the extent of dysfunction and outcome in patients suffering BBB disruption. Furthermore, we suggest that such procedures may correlate with cortical function and serve as follow-up measures in neurological patients. As observed in the present study, this might be especially useful for patients with TBI, but could also assist following recovery from other pathologies associated with increased BBB permeability (e.g., tumors, stroke, multiple sclerosis, and infectious diseases). It remains unclear as to what extent such dynamic measures of brain vascular bed permeability will be valuable in predicting the natural course of common neurological diseases.

## Figures and Tables

**Table 1:** Patient characteristics. Thirty-seven patients with TBI were enrolled in this study. Eighteen patients presented at our outpatient clinic with general complaints such as headaches and were included in the non-epileptic group. Nineteen patients presented with seizures and were included in the PTE patient group. sLORETA localization to Brodmann areas of abnormal EEG activity and enhancement is presented in parentheses.

#	Age	Symptoms	Abnormal enhancement	EEG interpretation
1	22	Acute stress reaction	No disruption	—
2	24	Cognitive impairment	Rt. frontal (47)	Normal
3	37	Headaches	No disruption	—
4	49	Headaches	No disruption	—
5	19	Headaches	—	Normal
6	25	Headaches	No disruption	Normal
7	27	Headaches	No disruption	—
8	17	Headaches	No disruption	—
9	25	Headaches	No disruption	Normal
10	17	Headaches	No disruption	—
11	22	Headaches	No disruption	—
12	46	Headaches	Rt. parietal (38)	—
13	13	Headaches	Rt. Parieto-occipital (18)	Normal (18Rt)
14	23	Headaches	—	Normal
15	13	Headaches	—	Normal
16	62	Headaches	No disruption	—
17	18	Headaches	No disruption	—
18	22	Motor Aphasia, Rt. Central Facialis	Lt. parietal (9)	Lt. fronto- parietal delta activity (9)
19	34	Behavioral Changes, Susp. Temporal seizures	Rt. temporal (21)	Rt. fronto-temporal delta activity (21)
20	29	Seizures	—	Normal
21	12	Seizures	—	Lt. temporal delta activity (47)
22	23	Seizures	Lt. parietal (40)	Lt. parietal epileptiform focus (47)
23	62	Seizures	Lt. parietal (2)	—
24	15	Seizures	Rt. parietal (40)	Rt. Temporo-parietal epileptiform activity (40)
25	10	Seizures	No disruption	Lt. frontal delta activitv 01 )
26	18	Seizures	Rt. temporo-occipital (37)	Rt. Temporal teta activitv (38)
27	18	Seizures	Rt. parietal (40)	—
28	37	Seizures	Rt. temporal (47)	Rt. Temporal epileptiform focus (47)
29	68	Seizures+ Rt. Hemiparesis	Lt. parietal (3)	—
30	17	Seizures	No disruption	Normal
31	15	Seizures	—	Lt. temporo-parietal delta activity (38)
32	49	Seizures	No disruption	Normal
33	28	Seizures	Lt. parietal (40)	Lt. temporo-parietal epileptiform activity (19)
34	17	Seizures	Lt. fronto-parietal (8)	Lt. frontal delta activity (47)
35	23	Seizures	Rt. parietal (40)	—
36	16	Seizures	Lt. parietal (40)	—
37	23	Seizures	—	Lt. fronto-temporal epileptiform activity (11)
